# Gami-Guibitang Attenuates Anxiety-like Behaviors and Modulates Hippocampal Synaptic Signaling in a Valproic Acid-Induced Mouse Model of Autism

**DOI:** 10.3390/brainsci16030259

**Published:** 2026-02-25

**Authors:** Ji Hye Yoon, Duk Jin Jung, Mikyung Kim, Young-Nam Kim, Minji Shim, Sung Youn Lee, Cheol Shin, Sangeun Im, Sungho Maeng, Jihwan Shin

**Affiliations:** 1 College of East-West Medical Science, Kyung Hee University, Yongin 17104, Republic of Korea; 2 College of Pharmacy, Dongduk Women’s University, Seoul 02707, Republic of Korea

**Keywords:** autism spectrum disorder, anxiety-like behavior, Gami-Guibitang, synaptic signaling, hippocampus

## Abstract

**Highlights:**

**What are the main findings?**

- Gami-Guibitang significantly attenuated anxiety-like behaviors in a valproic acid–induced mouse model of autism without causing behavioral suppression or cognitive impairment.
- Gami-Guibitang restored aberrant hippocampal synaptic signaling induced by prenatal valproic acid exposure, particularly within CREB–PI3K–Akt and GABAergic pathways.

**What are the implications of the main findings?**

- These findings suggest that Gami-Guibitang may selectively improve anxiety-related symptoms associated with autism spectrum disorder while preserving cognitive function.
- The modulation of hippocampal synaptic signaling highlights the potential of Gami-Guibitang as a multi-target therapeutic candidate for emotional dysregulation in neurodevelopmental disorders.

**Abstract:**

Background: Autism spectrum disorder (ASD) is a neurodevelopmental condition characterized by social deficits, repetitive behaviors, and heightened anxiety. Despite extensive research, effective interventions targeting core symptoms remain limited. Gami-Guibitang (GBT), a traditional herbal formula, has been clinically prescribed for anxiety-related symptoms and cognitive complaints, yet its effects on ASD-associated behavioral and molecular abnormalities have not been fully elucidated. Objective: This study aimed to evaluate the anxiolytic and neuroregulatory effects of GBT in a valproic acid (VPA)-induced ASD mouse model, focusing on behavioral outcomes and hippocampal synaptic protein expression. Methods: Pregnant C57BL/6N mice received a single intraperitoneal injection of VPA (500 mg/kg) at embryonic day 12.5. Male offspring were administered GBT (150 mg/kg, p.o.) twice daily for 4 weeks from postnatal day 21 (PND 21). These mice were behaviorally evaluated by the open-field test, elevated plus maze, marble-burying test, Y-maze, three-chamber social interaction test, and Morris water maze. Western blot analysis was conducted to examine hippocampal expression of phosphorylated and total CREB and GluR1, PI3K/Akt signaling components, as well as GABRA1 and GABRB1. Results: VPA-exposed offspring exhibited increased anxiety-like behaviors, altered repetitive behaviors, dysregulated exploratory activity, and impaired spatial learning, and reduced spontaneous alternation performance in the Y-maze. GBT reduced anxiety-like behaviors in the elevated plus maze and marble burying tests, partially improved spatial learning acquisition in the Morris water maze, and normalized excessive locomotor activity, without significantly affecting short-term working memory performance. At the molecular level, GBT significantly attenuated VPA-induced hyperphosphorylation of CREB, GluR1, PI3K, and Akt, indicating suppression of aberrant synaptic signaling rather than global enhancement. In addition, GBT increased GABRA1 expression toward control levels and enhanced GABRB1 expression beyond baseline, suggesting selective modulation of GABAergic receptor subunit composition rather than simple normalization. Conclusions: These findings provide preclinical evidence that GBT alleviates anxiety-like behavior and modulates hippocampal synaptic signaling disrupted by prenatal VPA exposure. By attenuating aberrant excitatory signaling and selectively regulating GABAergic receptor balance, GBT may represent a multi-target herbal candidate for modulating ASD-associated emotional dysregulation and domain-specific cognitive dysfunction, rather than acting as a broad cognitive enhancer.

## 1. Introduction

Autism spectrum disorder (ASD) is a neurodevelopmental disorder characterized by persistent social deficits and repetitive behaviors, and it is frequently accompanied by clinically significant anxiety [1]. Among these features, anxiety is one of the most disabling comorbid symptoms, affecting up to 40–50% of individuals with ASD and markedly reducing quality of life [1]. Globally, the reported prevalence of ASD has increased nearly fourfold over the past two decades. According to the Centers for Disease Control and Prevention (CDC), 1 in 36 children aged 8 years were identified with ASD in 2020 (reported in 2023), compared with 1 in 150 in 2000 [2]. In Korea, national insurance data indicated that annual medical expenditures for children with ASD under 10 years exceeded 190 billion KRW in 2022 [3]. The number of registered ASD patients has risen from approximately 20,000 in 2010 to over 50,000 in 2022, reflecting a steady increase in diagnostic prevalence and treatment demand [4]. These statistics underscore the substantial social and economic burden of ASD, highlighting the urgent need for therapeutic strategies targeting anxiety symptoms that exacerbate functional impairment and caregiver distress.

Despite extensive research, pharmacological treatments for ASD remain highly limited. Risperidone is among the few approved pharmacotherapies for ASD-associated behavioral symptoms and is primarily effective against irritability and hyperactivity rather than anxiety or social dysfunction [5,6]. Moreover, these drugs often induce adverse effects such as metabolic dysregulation, sedation, and weight gain [7], emphasizing the demand for safer, multi-target therapeutic alternatives.

At the molecular level, ASD pathology involves altered excitatory/inhibitory balance, synaptic plasticity impairment, and neuroinflammation [8,9]. Dysregulation of AMPA receptor subunits, including GluR1, has been implicated in synaptic plasticity abnormalities and has been discussed in recent reviews of ASD-related circuit and synapse dysfunction [10]. Abnormal neuronal development and cortical overconnectivity have also been observed in the prefrontal cortex of individuals with ASD [11]. In addition, disruption of GABAergic signaling contributes to neural network instability and anxiety-related phenotypes [12]. Emerging evidence also implicates altered stress responsivity and neuroimmune activation as key contributors to synaptic and behavioral abnormalities [13,14]. Collectively, these findings indicate that ASD involves alterations in synaptic plasticity–related signaling, including CREB-associated pathways, and glutamatergic–GABAergic synaptic imbalance, providing a mechanistic rationale for evaluating modulators of synaptic plasticity such as GBT in ASD-related anxiety.

Gami-Guibitang (GBT), a traditional herbal prescription modified from Guibi-tang, has been used clinically in the Republic of Korea, China, and Japan for managing anxiety, fatigue, impaired concentration, and memory-related symptoms, with reported clinical use and tolerability [15]. Guibi-tang consists of ten medicinal ingredients traditionally classified as tonifying and calming herbs, while GBT (also known as Kamikihito or Jia-Wei-Gui-Pi-Tang) is a modified formulation that incorporates additional components such as *Gardenia jasminoides* and *Bupleurum falcatum* [15]. Guibi-tang consists of 10 medicinal ingredients—Angelicae Radix, Longan Arillus, Ziziphi Semen, Polygalae Radix, Ginseng Radix, Astragali Radix, Atractylodis Rhizoma Alba, Poria Sclerotium cum Pini Radix, Aucklandiae Radix, and Glycyrrhizae Radix et Rhizoma. Gami-Guibitang (GBT), also known as Jia-Wei-Gui-Pi-Tang in Chinese medicine or Kamikihito in Japanese Kampo medicine, is a modified formulation of Guibi-tang that includes additional components such as *Gardenia jasminoides* and *Bupleurum falcatum* [15]. Although *Dimocarpus longan* and *Ziziphus jujuba* are both traditionally classified as nourishing herbs, they differ in medicinal parts and functional indications, with longan arillus primarily associated with emotional stabilization and fatigue, and Ziziphus seed traditionally linked to anxiolytic and sleep-related regulation. Experimental studies suggest that GBT exhibits multiple neuromodulatory actions, including reported effects on neurogenesis, neuroinflammatory regulation, and modulation of CREB-associated signaling [16,17]. Additionally, GBT has been reported to modulate social recognition–related behaviors in inflammation-based or genetic ASD models, rather than prenatal VPA-based models [18]. However, the effects of GBT have not been tested in ASD models induced by prenatal valproic acid (VPA) exposure. Given the rising prevalence of ASD, the social and economic burden of comorbid anxiety, and the limitations of currently available pharmacological options, this study aimed to evaluate the effects of GBT in a VPA-induced ASD mouse model, focusing on behavioral outcomes and hippocampal synaptic signaling pathways, including CREB phosphorylation, AMPA receptor–related signaling, PI3K/Akt activation, and GABAergic receptor subunit expression.

## 2. Materials and Methods

### 2.1. Reagents and Materials

Valproic acid (VPA; ≥99% purity) was obtained from Sigma-Aldrich (St. Louis, MO, USA).

The formula of Gami-Guibitang (GBT) comprises 14 medicinal herbs, as summarized in Table 1. GBT was prepared according to a conventional decoction procedure commonly used in clinical practice, as described previously [19]. Briefly, the mixed crude herbs were extracted in distilled water (1:10, *w*/*v*) for 2 h at 100 °C, filtered, and concentrated under reduced pressure. The extract was lyophilized to obtain a dried powder, which was stored at −20 °C until use. The ELISA kit used in biochemical assays is described in detail in Section 2.4.1, and the primary and secondary antibodies are described in Section 2.4.2 (Western Blotting). The Gami-Guibitang (GBT) extract used in this study was manufactured by Kyung Hee Medical Center (Seoul, Republic of Korea). A total of 49.2 g of crude herbal materials per prescription were extracted with distilled water under reflux for 3 h. Approximately 514 g of combined raw materials were processed under identical conditions, filtered through a 5 µm filter, concentrated under reduced pressure, and freeze-dried. The final dried extract weighed 178.91 g, corresponding to a yield of 34.81%. Raw herbal materials were authenticated and processed under standardized hospital quality control procedures to ensure consistency in botanical identity and preparation conditions.

### 2.2. Animals and Experimental Design

C57BL/6N mice were obtained from Daehan BioLink (Eumseong, Republic of Korea) and housed under controlled conditions (24 ± 1 °C; 55 ± 5% humidity; 12 h light/dark cycle) with free access to food and water.

**Figure 1:**
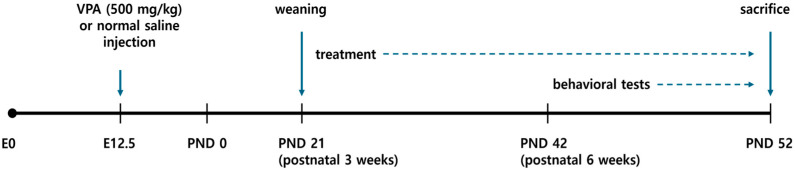
Schematic diagram of the experimental timeline for the GBT treatment on VPA-induced ASD model mice. Pregnant dams received a single intraperitoneal injection of VPA (500 mg/kg) on embryonic day 12.5 (E12.5). Male offspring were administered Gami-Guibitang (150 mg/kg per dose, p.o.) twice daily (total 300 mg/kg/day) for 4 weeks beginning at postnatal day 21. Behavioral assessments were performed from postnatal day 42 (PND 42) during the treatment period. Mice were sacrificed at PND 52 for biochemical analyses.

Pregnant mice received a single intraperitoneal injection of valproic acid (VPA; 500 mg/kg in saline, 10 mL/kg) on embryonic day 12.5 (E12.5) to establish the autism spectrum disorder (ASD) model [20]. Control dams received the same volume of normal saline. After birth, pups were weaned on postnatal day 21, and only male offspring were used for experiments to avoid hormonal variability. Offspring were derived from six independent litters per prenatal treatment condition. To minimize potential litter effects, male pups were distributed across experimental groups after weaning, and no more than two pups from the same litter were assigned to the same treatment group whenever possible. Each experimental group therefore included animals originating from multiple independent litters. Male offspring were randomly divided into four groups: VC (vehicle control), normal mice administered distilled water (DW); VGBT (GBT-treated control), normal mice administered Gami-Guibitang (GBT; 150 mg/kg, p.o.); ASD (VPA-exposed), offspring prenatally exposed to VPA and administered DW; and AGBT (VPA + GBT-treated), offspring prenatally exposed to VPA and administered GBT (150 mg/kg, p.o.). GBT was dissolved in distilled water and administered orally at 150 mg/kg per dose, twice daily (total 300 mg/kg/day), for 4 weeks beginning at PND 21. The dosage and treatment duration were selected based on previous pharmacological reports evaluating behavioral outcomes [16,17,18]. Behavioral assessments were initiated after 3 weeks of GBT treatment (PND 42) and conducted during the treatment period, after which mice were sacrificed at PND 52 for biochemical analyses using blood and hippocampal tissue. All assessments were performed by investigators blinded to group assignments. Each experimental group consisted of six animals (n = 6 per group) for behavioral analyses. The same cohort of animals (n = 6 per group) was used for biochemical analyses unless otherwise specified. The overall experimental timeline, including prenatal VPA exposure, postnatal GBT administration, and behavioral assessments, is illustrated in Figure 1. All experimental procedures were approved by the Institutional Animal Care and Use Committee of Kyung Hee University (Approval No. KHGASP-23-178) and were performed in accordance with the NIH Guide for the Care and Use of Laboratory Animals.

### 2.3. Behavioral Tests

Behavioral tests were conducted to evaluate anxiety-like behavior, repetitive behavior, social interaction, and cognitive performance in VPA-exposed offspring and age-matched controls. All tests were performed between 09:00 and 17:00 in a sound-attenuated room under controlled lighting (20–30 lux). Mice were habituated to the testing room for at least 30 min before each test. Apparatus were cleaned with 70% ethanol between sessions to remove olfactory cues. Behavioral data were analyzed using SMART 3.0 software (Panlab, Harvard Apparatus, Barcelona, Spain).

#### 2.3.1. Open Field Test (OFT)

The OFT was used to evaluate locomotor activity and anxiety-like behavior [21]. Mice were placed individually in a square Plexiglas arena (50 × 50 × 40 cm) illuminated at 20 lux and allowed to explore freely for 30 min. Total distance traveled, time spent in the center zone (25 × 25 cm), and freezing duration were recorded.

#### 2.3.2. Elevated Plus Maze (EPM)

The EPM was used to assess anxiety-related behavior [21]. The apparatus consisted of two open arms and two closed arms (30 × 5 × 15 cm) arranged in a plus shape and elevated 40 cm above the floor. Each mouse was placed on the central platform facing an open arm and observed for 5 min. The time spent in and the number of entries into open arms were measured.

#### 2.3.3. Marble Burying Test (MBT)

The MBT was used to assess repetitive and anxiety-related behaviors [22]. Mice were placed individually in a cage (35 × 15 × 12 cm) containing 5 cm of bedding with 12 evenly spaced glass marbles. After 10 min, the number of marbles buried (≥2/3 covered) was counted.

#### 2.3.4. Y-Maze Test

The Y-maze test was used to evaluate working memory and exploratory behavior [23]. The apparatus consisted of three identical arms (30 × 5 × 15 cm) positioned at 120° angles. Mice were placed at the end of one arm and allowed to explore freely for 5 min. The percentage of spontaneous alternation was calculated as follows: % alternation = {number of alternations/(total arm entries − 2)} × 100, and higher alternation rates indicated better working memory performance.

#### 2.3.5. Three-Chamber Social Interaction Test

The three-chamber social interaction test was used to assess sociability and social preference behavior [22]. The apparatus consisted of three interconnected chambers (17 × 13 × 25 cm each), with the central chamber serving as a neutral exploration zone and the two side chambers designated for stimulus presentation. Each side chamber contained a wire cup (diameter: 5 cm) with vertical bars that allowed sensory interaction while preventing direct contact. The test consisted of three sequential phases. In the habituation phase, mice were allowed to freely explore all three empty chambers for 10 min to acclimate to the apparatus. In the object exploration phase, identical empty wire cups were placed in both side chambers, and mice were allowed to explore for 10 min to assess baseline exploratory behavior toward non-social stimuli. Following a 10 min rest period, the sociability phase was conducted, during which an unfamiliar mouse (Stranger 1) was placed under a wire cup in one side chamber, while an empty cup remained in the opposite chamber. Mice were allowed to explore freely for 10 min, and the time spent in each zone (object, center, and stranger zones) was recorded and analyzed as an index of social approach behavior. The social novelty phase was not included in the present study.

#### 2.3.6. Morris Water Maze (MWM)

The MWM was used to evaluate spatial learning and memory [24]. The apparatus consisted of a circular pool (diameter 180 cm, height 45 cm) filled with opaque water maintained at 22 ± 1 °C. A hidden platform (10 cm in diameter) was submerged 1 cm below the water surface in one quadrant. Mice underwent four acquisition trials per day for five consecutive days. A visual platform was used on day 1 and 2, and the location of the platform was hidden on day 3 to 5. The starting position varied across trials. If a mouse failed to locate the platform within 60 s, it was gently guided to the platform and allowed to remain there for 10 s. For the Morris water maze, statistical analyses were limited to acquisition-phase escape latency, as no probe trial was performed.

### 2.4. Biochemical Analyses

#### 2.4.1. Enzyme-Linked Immunosorbent Assay (ELISA)

Serum tumor necrosis factor-alpha (TNF-α) levels were quantified using a commercial ELISA kit (#MBS825075, MyBioSource, San Diego, CA, USA) according to the manufacturer’s protocol. Whole blood was collected via the abdominal vena cava and centrifuged at 3000 rpm for 15 min at 4 °C to obtain serum. Absorbance was measured at 450 nm using a microplate reader (Molecular Devices, San Jose, CA, USA), and TNF-α concentrations were calculated from a standard curve and expressed as pg/mL.

#### 2.4.2. Western Blotting

Western blotting was performed using a standard protocol. Briefly, hippocampal tissues were dissected and homogenized in RIPA buffer containing protease and phosphatase inhibitors. Protein concentrations were determined using the BCA assay. Equal amounts of protein (15–30 μg) were separated by SDS–PAGE, transferred onto PVDF membranes, and blocked with 5% skim milk in TBS–T. Membranes were incubated overnight at 4 °C with primary antibodies against BDNF, GABRA1, GABRB1, phospho-CREB (p-CREB) and total CREB, phospho-GluR1 (p-GluR1) and total GluR1, phospho-PI3K (p-PI3K) and total PI3K, and phospho-Akt (p-Akt) and total Akt, with GAPDH or β-actin used as loading controls. After washing, membranes were incubated with HRP-conjugated secondary antibodies for 1 h at room temperature. Protein bands were detected using an enhanced chemiluminescence (ECL) system, and densitometric analysis was performed using ImageJ software (version 1.54g, NIH, Bethesda, MD, USA).

### 2.5. Statistical Analysis

All data were expressed as mean ± standard error of the mean (SEM) and analyzed using IBM SPSS Statistics version 26 (IBM Corp., Armonk, NY, USA). One-way ANOVA was used for single-time-point group comparisons, followed by post hoc testing (LSD or Games–Howell, as appropriate). For repeated-measures data including body weight, food intake, and Morris water maze acquisition trials, repeated-measures ANOVA was performed to evaluate within-group effects (time), between-group effects (treatment), and time × treatment interactions. For datasets that violated the assumption of homogeneity of variance (Levene’s test), Welch ANOVA was applied, followed by the Games–Howell post hoc test. Statistical significance was set at *p* < 0.05, *p* < 0.01, and *p* < 0.001.

## 3. Results

### 3.1. Crooked Tail Deformity in Prenatal VPA-Exposed Mice

**Figure 2:**
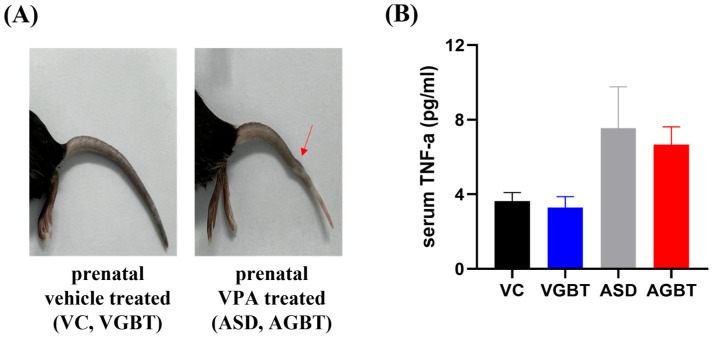
Effect of prenatal VPA exposure in mice. (**A**) Representative tail malformations (arrow) observed in mice prenatally exposed to VPA. (**B**) Serum TNF-α levels measured at PND52 following the 4-week GBT treatment period, showing no statistically significant differences among groups. One-way ANOVA: F(3,20) = 2.9, *p* = 0.062; data are presented as mean ± SEM. VC (vehicle control): prenatal normal saline + 4 weeks DW p.o.; VGBT (vehicle, GBT): prenatal normal saline + 4 weeks GBT (150 mg/kg, p.o.); ASD (VPA-exposed): prenatal VPA (500 mg/kg) + 4 weeks DW p.o.; AGBT (VPA-exposed + GBT): prenatal VPA (500 mg/kg) + 4 weeks GBT (150 mg/kg, p.o.).

Mice prenatally exposed to VPA exhibited distinct tail malformations that were absent in vehicle-treated control mice (Figure 2A). Serum TNF-α levels measured at PND52 showed a non-significant trend toward elevation in VPA-exposed mice (ASD, AGBT) compared with vehicle-treated controls (F(3,20) = 2.9, *p* = 0.062).

### 3.2. Effects of GBT on Growth Parameters and Organ Weights

**Figure 3:**
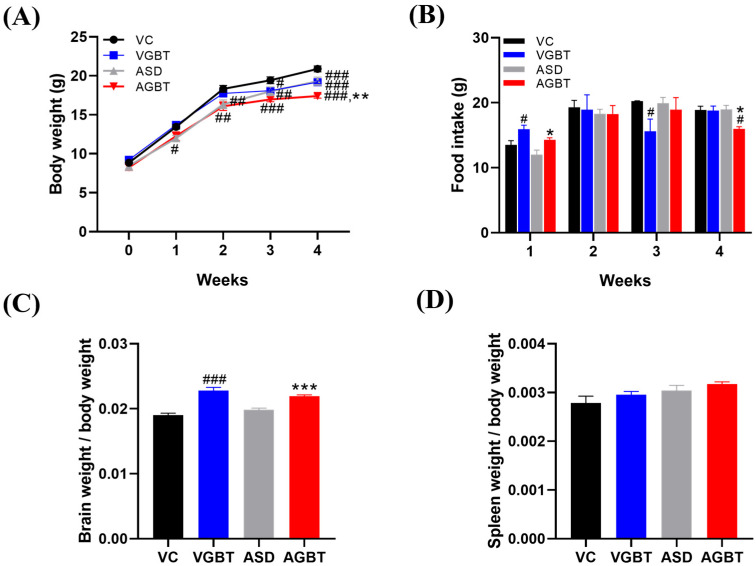
Effects of GBT on growth parameters and organ weights in VPA-exposed offspring. (**A**) Changes in body weight during the 4-week treatment period. (**B**) Weekly food intake during the experimental period. (**C**) Brain-to-body weight ratio after 4 weeks of GBT treatment. (**D**) Spleen-to-body weight ratio after 4 weeks of GBT treatment. Data are presented as mean ± SEM. For panels (**A**,**B**), data were analyzed using repeated-measures ANOVA followed by LSD post hoc test. For panels (**C**,**D**), data were analyzed using one-way ANOVA followed by LSD post hoc test. # *p* < 0.05, ## *p* < 0.01, ### *p* < 0.001 vs. VC; * *p* < 0.05, ** *p* < 0.01, *** *p* < 0.001 vs. ASD. VC: prenatal normal saline + DW p.o.; VGBT: prenatal normal saline + GBT (150 mg/kg, p.o.); ASD: prenatal VPA (500 mg/kg) + DW p.o.; AGBT: prenatal VPA (500 mg/kg) + GBT (150 mg/kg, p.o.).

Body weight changed significantly over time in all groups, with significant main effects of time (F(2.4,48.6) = 1083, *p* < 0.001), group (F(3,20) = 8.2, *p* < 0.001), and a significant time × group interaction (F(7.3,48.6) = 5.3, *p* < 0.001). Post hoc analysis revealed that ASD offspring exhibited significantly lower body weight compared with VC mice (VC vs. ASD, *p* = 0.004), whereas GBT treatment did not significantly restore body weight in VPA-exposed mice (ASD vs. AGBT, *p* = 0.217), despite an overall group × time interaction (Figure 3A). Weekly food intake did not differ significantly among groups, as repeated-measures ANOVA revealed a significant main effect of time but no significant main effect of group, although a significant time × group interaction was observed (Figure 3B). Brain-to-body weight ratio differed significantly among groups (one-way ANOVA, F(3,20) = 29.3, *p* < 0.001), with VGBT mice showing a significantly higher ratio compared with VC (*p* < 0.001), and AGBT mice exhibiting a significantly increased ratio compared with ASD offspring (*p* < 0.001) (Figure 3C). Spleen-to-body weight ratio did not differ significantly among groups (one-way ANOVA, F(3,20) = 2.781, *p* = 0.068) (Figure 3D). These findings indicate that prenatal VPA exposure impaired overall growth trajectory without substantially altering food intake, and that GBT administration did not significantly restore body weight, while selectively normalizing the brain-to-body weight ratio.

### 3.3. GBT Reduces Anxiety-like Behaviors and Behavioral Suppression

**Figure 4:**
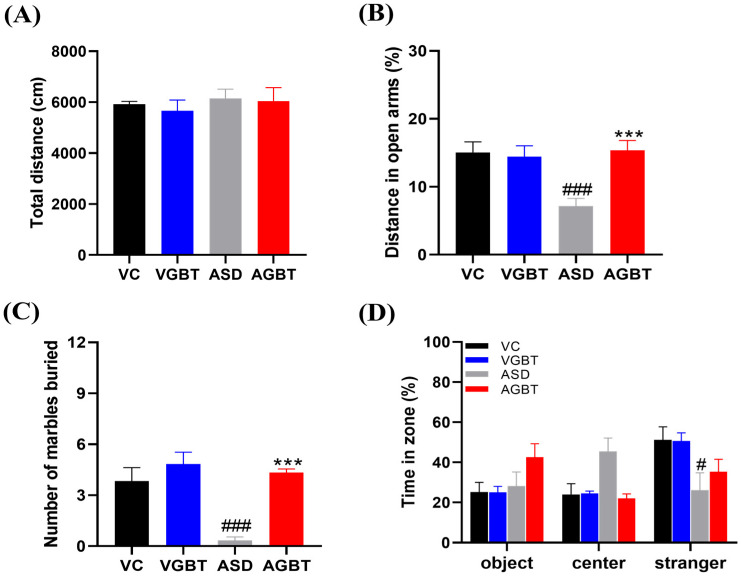
Effects of 4-week GBT treatment on locomotion, anxiety-like, repetitive, and social behaviors in prenatal VPA-exposed mice. (**A**) Total distance traveled in the open-field test (F(3,20) = 0.289, *p* = 0.833). (**B**) Percentage of distance traveled in the open arms of the elevated plus maze (F(3,20) = 7.404, *p* = 0.002). (**C**) Number of marbles buried in the marble-burying test (F(3,20) = 13.761, *p* < 0.001). (**D**) Time spent (%) in the object, center, and stranger zones during the sociability phase of the three-chamber social interaction test. One-way ANOVA showed no significant group differences in the object zone (F(3,20) = 2.172, *p* = 0.123) or center zone (Welch ANOVA: F(3,9.6) = 3.317, *p* = 0.067), whereas a significant group effect was observed in the stranger zone (F(3,20) = 3.474, *p* = 0.035). Paired *t*-test analysis comparing time spent in the object and stranger zones within each group revealed significant social preference in the VC (*p* = 0.050) and VGBT (*p* = 0.015) groups, but not in the ASD (*p* = 0.894) or AGBT (*p* = 0.599) groups. Data are presented as mean ± SEM. # *p* < 0.05, ### *p* < 0.001 vs. VC *** *p* < 0.001 vs. ASD. One-way ANOVA followed by LSD post hoc test unless otherwise indicated. VC: prenatal normal saline + DW p.o.; VGBT: prenatal normal saline + GBT (150 mg/kg, p.o.); ASD: prenatal VPA (500 mg/kg) + DW p.o.; AGBT: prenatal VPA (500 mg/kg) + GBT (150 mg/kg, p.o.).

In the open-field test, total distance traveled did not differ significantly among groups (F(3,20) = 0.289, *p* = 0.833), indicating that neither prenatal VPA exposure nor GBT administration affected baseline locomotor activity (Figure 4A). Thus, subsequent behavioral alterations were not attributable to changes in general locomotion. In the elevated plus maze, ASD mice exhibited a significant reduction in the percentage of distance traveled in the open arms compared with VC mice (*p* < 0.001), reflecting increased anxiety-like behavior (Figure 4B). GBT treatment significantly increased open-arm exploration relative to ASD mice (*p* < 0.001), indicating attenuation of anxiety-like behavior induced by prenatal VPA exposure. In the marble-burying test, ASD mice buried significantly fewer marbles than VC mice (*p* < 0.001), suggesting behavioral suppression associated with heightened anxiety or avoidance rather than enhanced repetitive behavior (Figure 4C). GBT administration significantly increased the number of buried marbles compared with ASD mice (*p* < 0.001), indicating partial restoration of marble-burying behavior toward control levels. In the social interaction test, one-way ANOVA revealed no significant group differences in time spent in the object zone (F(3,20) = 2.172, *p* = 0.123) or center zone (Welch ANOVA: F(3,9.6) = 3.317, *p* = 0.067). In contrast, a significant group effect was observed for time spent in the stranger zone (F(3,20) = 3.474, *p* = 0.035), with ASD mice showing reduced stranger-zone exploration compared with VC mice (*p* = 0.014) (Figure 4D). Social preference analysis using paired t-tests revealed significant differences between object and stranger zones in the VC (*p* = 0.050) and VGBT (*p* = 0.015) groups, whereas no significant preference was observed in the ASD (*p* = 0.894) or AGBT (*p* = 0.599) groups (Figure 4D).

### 3.4. GBT Partially Improves Social Interaction Deficits

**Figure 5:**
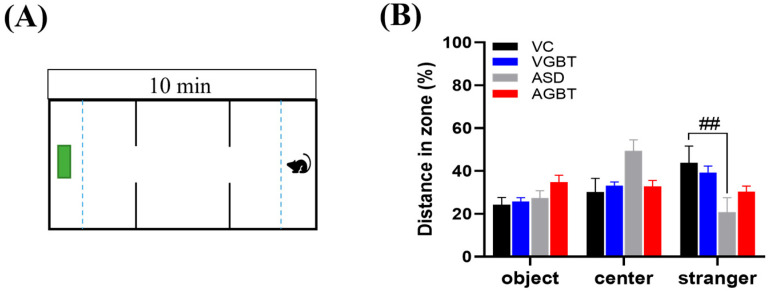
Effects of GBT on social interaction behavior in prenatal VPA-exposed offspring. (**A**) Schematic diagram of the three-chamber apparatus used for the sociability test. (**B**) Time spent (%) in the object, center, and stranger zones during the sociability phase. ASD mice showed a significant reduction in time spent in the stranger zone compared with VC mice, while GBT treatment significantly increased stranger zone exploration relative to ASD mice. F(3,20) = 3.361, *p* = 0.039. Data are presented as mean ± SEM. ## *p* < 0.01 vs. VC. One-way ANOVA followed by LSD post hoc test. The dashed lines indicate the boundaries between the object, center, and stranger zones, and the green box represents the object location.

VPA-exposed offspring exhibited reduced sociability in the three-chamber social interaction test, consistent with the alterations observed in Figure 4D (Figure 5A). During the sociability phase, ASD mice showed a significant reduction in time spent in the stranger zone compared with VC mice (*p* = 0.039), indicating diminished social approach behavior (Figure 5B). Instead, ASD mice displayed a relative shift toward the object zone, reflecting avoidance of social stimuli. GBT treatment significantly increased the time spent in the stranger zone compared with untreated ASD mice (## *p* < 0.01), suggesting partial improvement in social approach behavior. Although the magnitude of recovery did not fully reach that of VC mice, AGBT offspring exhibited a spatial exploration pattern closer to controls, indicating partial restoration rather than complete normalization of sociability (Figure 5B).

### 3.5. GBT Improves Learning and Memory Performance

**Figure 6:**
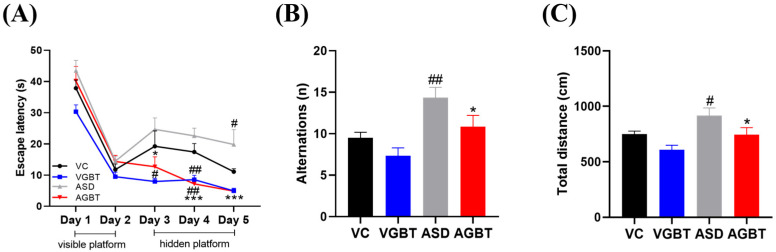
Effects of GBT on learning and memory performance in VPA-exposed offspring. (**A**) Escape latency across training days in the Morris Water Maze. (**B**) Number of alternations in the Y-maze. (**C**) Total distance traveled in the Y-maze. Data are presented as mean ± SEM. # *p* < 0.05, ## *p* < 0.01 vs. VC; * *p* < 0.05, *** *p* < 0.001 vs. ASD. Repeated-measures ANOVA was applied for panel (**A**), and one-way ANOVA followed by LSD post hoc test was applied for panels (**B**,**C**).

In the Morris water maze, escape latency across training days showed significant within-group effects (F(4,80) = 69.594, *p* < 0.001) and between-group differences (F(3,20) = 21.716, *p* < 0.001), whereas the group × time interaction did not reach significance (F(12,80) = 1.575, *p* = 0.116) (Figure 6A). ASD mice demonstrated prolonged escape latencies during hidden-platform trials, reflecting deficits in spatial learning. GBT-treated ASD mice displayed improved acquisition performance, with a significant reduction in escape latency compared with ASD mice during the acquisition phase (*p* < 0.001 vs. ASD), indicating improved learning efficiency rather than full restoration of spatial memory. In the Y-maze test, the number of alternations differed significantly among groups (F(3,20) = 7.1, *p* = 0.002), with ASD mice showing a significant reduction compared with VC (*p* = 0.006), indicating impaired short-term working memory (Figure 6B). GBT treatment significantly increased alternation performance relative to ASD mice (*p* = 0.036), suggesting partial improvement in working memory-related performance. Total distance traveled in the Y-maze differed significantly among groups (F(3,20) = 5.488, *p* = 0.006), with ASD mice showing increased locomotor activity relative to VC (*p* = 0.041), reflecting altered exploratory behavior following prenatal VPA exposure (Figure 6C). GBT administration significantly reduced this hyperactivity compared with ASD mice (*p* = 0.035), indicating partial normalization of exploratory behavior. Collectively, these findings indicate that GBT improves acquisition-phase spatial learning and working memory performance in VPA-exposed mice, while partially normalizing exploratory behavior without fully restoring spatial learning to control levels.

### 3.6. GBT Modulates Synaptic Plasticity–Related Signaling in the Hippocampus

**Figure 7:**
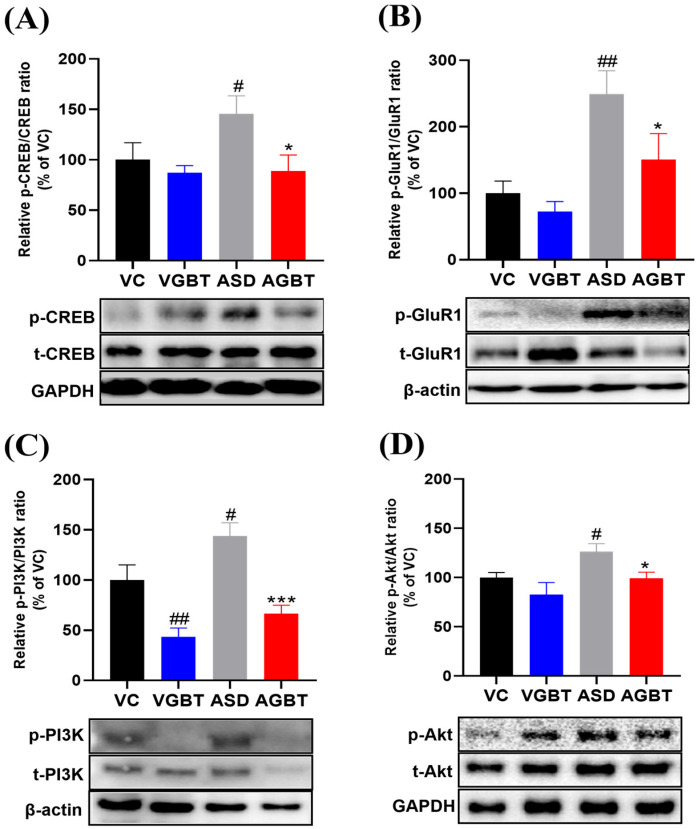
Effects of GBT on hippocampal synaptic plasticity–related signaling in VPA-exposed offspring. (**A**) Relative p-CREB/t-CREB expression. (**B**) Relative p-GluR1/GluR1 expression. (**C**) Relative p-PI3K/t-PI3K expression. (**D**) Relative p-Akt/t-Akt expression. Representative Western blots corresponding to each graph are shown below. Data are presented as mean ± SEM. # *p* < 0.05, ## *p* < 0.01 vs. VC; * *p* < 0.05, *** *p* < 0.001, vs. ASD. One-way ANOVA followed by LSD post hoc test was applied. When the assumption of homogeneity of variance was violated, Welch ANOVA followed by the Games–Howell post hoc test was used.

**Figure 8:**
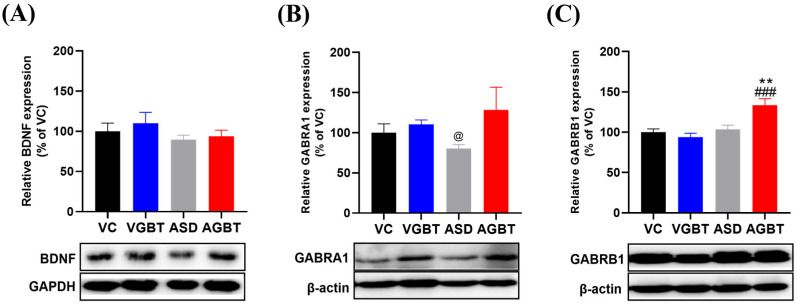
Effects of GBT on hippocampal neurotrophic and GABAergic receptor expression in VPA-exposed offspring. (**A**) Relative BDNF expression. (**B**) GABAA receptor α1 (GABRA1) expression. (**C**) GABAA receptor β1 (GABRB1) expression. Representative Western blots are shown below each graph. Data are presented as mean ± SEM. ### *p* < 0.001 vs. VC; ** *p* < 0.01 vs. ASD; @ *p* < 0.05 vs. VGBT. One-way ANOVA followed by LSD post hoc test was applied. For GABRA1 expression, Welch ANOVA followed by the Games–Howell post hoc test was used due to violation of homogeneity of variance.

Hippocampal analysis revealed that GBT regulated multiple signaling pathways disrupted by prenatal VPA exposure. First, CREB phosphorylation was significantly increased in ASD mice, as indicated by an elevated p-CREB/t-CREB ratio compared with VC (Figure 7A, F(3,20) = 3.379, *p* = 0.039), consistent with previous reports suggesting that prenatal VPA exposure is associated with aberrant CREB signaling involved in synaptic plasticity regulation. GBT administration significantly reduced this hyperactivation, as evidenced by a significant decrease in p-CREB/t-CREB ratio compared with ASD mice (Figure 7A, *p* = 0.014). AMPA receptor-related signaling also showed clear alterations. VPA exposure markedly increased hippocampal p-GluR1/GluR1 levels relative to VC (Figure 7B, F(3,20) = 7.224, *p* = 0.002), indicating enhanced excitatory drive. GBT effectively attenuated this upregulation, as p-GluR1/GluR1 levels were significantly reduced in AGBT mice compared with ASD mice (Figure 7B, *p* = 0.026). In addition to CREB–AMPAR signaling, the PI3K/Akt pathway exhibited VPA-induced dysregulation. ASD mice showed significantly increased p-PI3K/t-PI3K and p-Akt/t-Akt expression relative to VC (Figure 7C,D; p-PI3K: F(3,20) = 14.016, *p* < 0.001; p-Akt: F(3,20) = 4.731, *p* = 0.012), indicating aberrant hyperactivation of this plasticity-related signaling pathway at the phosphorylation level. Notably, GBT administration in non-VPA–exposed mice slightly reduced basal PI3K phosphorylation compared with VC, without detectable behavioral consequences. GBT treatment significantly attenuated the VPA-induced hyperactivation of PI3K and Akt phosphorylation, as evidenced by significantly reduced p-PI3K/t-PI3K and p-Akt/t-Akt ratios in AGBT mice compared with ASD mice (Figure 7C,D; *p* < 0.001 and *p* = 0.030, respectively). In contrast to these signaling changes, total BDNF expression did not differ significantly among groups (Figure 8A, F(3,20) = 0.814, *p* = 0.501), suggesting that basal neurotrophic expression was not markedly altered under the present experimental conditions. GABAergic receptor expression showed selective modulation. The α1 subunit (GABRA1) was reduced in ASD mice, and GBT treatment significantly increased its expression compared with ASD mice (Figure 8B, Welch ANOVA: F(3,10.381) = 5.451, *p* = 0.017), indicating partial restoration of GABAergic receptor balance. The β1 subunit (GABRB1) did not differ significantly between VC and ASD mice; however, GBT treatment significantly increased GABRB1 expression compared with ASD mice (Figure 8C, F(3,20) = 9.424, *p* < 0.001).

Together, these molecular findings demonstrate that GBT attenuates VPA-induced hyperactivation of multiple synaptic regulatory pathways—including CREB phosphorylation, AMPAR activity, and PI3K/Akt signaling—while selectively modulating GABA receptor subunit composition in the hippocampus. These molecular changes were observed alongside improvements in anxiety-like and social behavioral domains, supporting an association between restoration of synaptic signaling homeostasis and behavioral regulation in VPA-exposed mice.

## 4. Discussion

This study demonstrates that Gami-Guibitang (GBT), a multi-herbal prescription traditionally used for anxiety and cognitive weakness, attenuates several ASD-relevant behavioral abnormalities and modulates hippocampal synaptic signaling disrupted by prenatal valproic acid (VPA) exposure. GBT exerted corrective effects primarily on anxiety-like and repetitive behavioral domains without affecting general locomotor activity. At the molecular level, behavioral recovery was accompanied by normalization of hippocampal CREB phosphorylation, modulation of AMPA receptor-related signaling, and attenuation of aberrant PI3K/Akt hyperactivation, consistent with prior evidence linking CREB and glutamatergic dysregulation to ASD pathology [8,10]. Collectively, these results suggest that GBT mitigates ASD-like phenotypes primarily through synaptic plasticity–related pathways rather than peripheral mechanisms.

### 4.1. GBT Alleviates Anxiety-like and Repetitive Behaviors

Prenatal VPA exposure is widely used to model ASD-like behavioral abnormalities, producing enhanced anxiety, altered repetitive behavior, and reduced sociability in rodents [20]. The present findings confirmed key aspects of these phenotypes: ASD mice exhibited elevated anxiety in the elevated plus maze and reduced marble-burying behavior, consistent with anxiety-related behavioral suppression rather than enhanced compulsive repetition, aligning with prior reports [21,22]. Chronic GBT administration significantly improved anxiety-like behavior and normalized marble-burying behavior without altering locomotor activity in the open field test, indicating a true anxiolytic effect rather than sedation. These results are consistent with clinical and experimental reports showing that GBT alleviates anxiety and stress-induced behavioral disturbances [19]. Thus, GBT appears to exert selective behavioral benefits by improving emotional regulation and anxiety-related repetitive behaviors associated with ASD.

### 4.2. GBT Improves Learning and Partially Restores Cognitive Performance

Impairments in spatial learning and memory are commonly observed in VPA-based ASD models [20,25]. In this study, ASD mice exhibited prolonged escape latency during Morris water maze acquisition, consistent with spatial learning deficits reported previously [25]. GBT treatment significantly improved acquisition performance but did not fully restore long-term spatial memory, indicating that GBT enhances learning efficiency rather than complete memory consolidation. Because a probe trial was not conducted, the observed reduction in escape latency should be interpreted as improved learning during acquisition rather than confirmed enhancement of spatial memory retention. In the Y-maze, ASD mice showed reduced alternation frequency, indicating impaired short-term working memory [23]. GBT treatment partially restored alternation performance, suggesting normalization of exploratory strategy rather than restoration of working memory. This domain-specific effect aligns with the traditional use of GBT for cognitive fatigue and impaired concentration [19] and supports previous evidence that GBT modulates learning-related signaling pathways [16,17].

Although ASD mice exhibited increased total distance traveled in the Y-maze, this finding does not necessarily indicate enhanced functional exploration. Rather, increased locomotor activity in this context may reflect disorganized or repetitive exploratory patterns commonly observed in VPA-induced models. In contrast, reduced marble burying likely represents context-dependent behavioral suppression under anxiogenic conditions rather than a general locomotor deficit. Importantly, decreased burying was not accompanied by reduced overall mobility in the open-field test, arguing against a simple locomotor confound. Together, these findings indicate domain-specific dysregulation of exploratory and anxiety-related behaviors rather than uniform alterations in locomotion.

### 4.3. Peripheral Inflammation Is Not a Primary Target of GBT

Neuroimmune dysfunction, including elevated pro-inflammatory cytokines such as TNF-α, has been frequently reported in ASD [9,14]. In the present study, VPA-exposed offspring showed high variability in serum TNF-α, with ANOVA indicating a non-significant trend of increased serum concentration. GBT did not significantly alter serum TNF-α levels in ASD mice, suggesting that peripheral TNF-α alone may not represent a primary mechanism underlying the observed behavioral effects under the present experimental conditions. This aligns with the interpretation that the behavioral improvements induced by GBT arise predominantly from central neural mechanisms rather than systemic anti-inflammatory actions. Thus, while peripheral cytokines may contribute to ASD pathology, they do not appear to represent a primary mechanism underlying the therapeutic effects of GBT in the present model. Because only serum TNF-α was assessed, brain-specific inflammatory markers or microglial activation states were not evaluated, and therefore central neuroinflammatory involvement cannot be excluded.

### 4.4. GBT Modulates Hippocampal CREB–AMPAR Signaling

CREB phosphorylation and AMPA receptor trafficking are critical regulators of synaptic plasticity, emotional learning, and excitatory–inhibitory balance [10,26]. Dysregulated CREB activity has been reported in ASD and contributes to impaired synaptic function and anxiety-related behavior. In the present study, VPA exposure increased p-CREB expression in the hippocampus, consistent with previous findings implicating aberrant CREB-dependent transcription in ASD-like phenotypes [26,27]. Importantly, although CREB and PI3K/Akt signaling are classically associated with synaptic plasticity and memory formation, excessive or dysregulated activation of these pathways under neurodevelopmental stress conditions may reflect maladaptive plasticity rather than functional enhancement. In the VPA-induced ASD model, elevated phosphorylation levels likely represent aberrant or compensatory hyperactivation of synaptic signaling pathways, which may contribute to network instability and anxiety-related phenotypes. Therefore, attenuation of p-CREB and PI3K/Akt hyperphosphorylation by GBT should be interpreted as restoration of synaptic signaling balance rather than suppression of physiological plasticity. GBT treatment normalized the VPA-induced elevation of p-CREB levels rather than suppressing basal CREB activity and attenuated aberrant GluR1 phosphorylation without altering total GluR1 expression, suggesting restoration of synaptic signaling balance. The observed normalization of CREB and PI3K/Akt hyperphosphorylation may reflect the multi-component pharmacological profile of GBT. Bioactive compound classes present in its constituent herbs, including flavonoids, saponins, and phenolic compounds, have been reported to influence CREB-dependent transcription and PI3K/Akt-related plasticity pathways. These properties offer a plausible mechanistic basis linking the phytochemical profile of GBT to the normalization of hippocampal signaling observed in the present model. Several constituent herbs of GBT, including Astragalus membranaceus, have been reported to possess neuroprotective and synaptic modulatory properties, which may contribute to the observed normalization of CREB–AMPAR signaling. These combined actions likely contribute to restoration of synaptic signaling homeostasis and underlie the behavioral improvements observed.

### 4.5. Limitations

This study focused on hippocampal pathways, but other regions critical for ASD-related behaviors, such as the prefrontal cortex, amygdala, and hypothalamus, were not examined. These regions regulate emotional and social processing, including stress-related circuitry and neuroendocrine integration through corticotropin-releasing hormone (CRH) and oxytocin signaling pathways [28,29,30]. Future work should investigate whether GBT affects these circuits. In addition, downstream synaptic markers such as BDNF, PSD-95, and synaptophysin were not assessed to avoid excessive molecular profiling in this initial mechanistic study [27,31]. Finally, because GBT is a multi-component formula, identifying specific bioactive constituents remains an important direction for future pharmacological characterization. Although the extract was prepared under standardized hospital conditions with a defined extraction yield, comprehensive chemical fingerprinting (e.g., HPLC/UPLC profiling) and marker compound quantification were not performed in the present study Because a probe trial was not conducted in the Morris water maze paradigm, long-term spatial memory retention could not be definitively evaluated under the present experimental design. Although offspring were balanced across independent litters, statistical analyses were conducted at the individual animal level rather than incorporating litter as a nested random factor. Future studies applying litter-based mixed-effects models would further enhance statistical rigor.

## 5. Conclusions

In summary, this study demonstrates that Gami-Guibitang (GBT) exerts selective behavioral and molecular benefits in a VPA-induced autism spectrum disorder (ASD) mouse model. First, GBT did not significantly reduce serum TNF-α levels, suggesting that peripheral inflammation is unlikely to represent a primary therapeutic target of this formula. GBT also did not significantly counteract prenatal VPA-induced reductions in body weight, indicating limited effects on overall somatic growth under the present experimental conditions.

In contrast, GBT normalized the brain-to-body weight ratio, which was reduced by prenatal VPA exposure, indicating a potential regulatory effect on brain–body growth balance under neurodevelopmental stress conditions. Behaviorally, GBT reduced anxiety-like responses and normalized marble-burying behavior, while partially improving social approach behavior as assessed by the three-chamber sociability test, suggesting meaningful improvement in emotional and social deficits associated with ASD.

GBT additionally improved spatial learning during acquisition trials in the Morris water maze, whereas changes in Y-maze alternation patterns likely reflect improved regulation of exploratory strategy rather than full restoration of short-term working memory. This indicates domain-specific cognitive efficacy. At the molecular level, ASD mice exhibited elevated hippocampal p-CREB, a marker of dysregulated synaptic plasticity, and GBT effectively normalized this phosphorylation and attenuated VPA-induced hyperactivation of AMPAR- and PI3K/Akt-related signaling pathways, supporting restoration of synaptic signaling homeostasis.

Collectively, these findings indicate that GBT primarily acts on central synaptic regulatory pathways, rather than peripheral inflammatory mechanisms, to ameliorate anxiety-like behavior, social deficits, and spatial learning impairments in ASD-like conditions. GBT may therefore represent a promising multi-target herbal candidate for modulating emotional and cognitive dysfunction associated with ASD-like neurodevelopmental conditions.

## Figures and Tables

**Table 1:** Composition of Gami-Guibitang. All crude herbal materials were authenticated and supplied through the standardized procurement system of Kyung Hee Medical Center prior to decoction.

Herb Name	Amount (g)
*Angelica gigas* Nakai	4
*Dimocarpus longan* Lour.	4
*Ziziphus jujuba* var. *spinosa* Hu	4
*Atractylodes macrocephala* Koidz.	4
*Poria cocos* (Schw.) Wolf	4
*Panax ginseng* C.A. Meyer	4
*Astragalus membranaceus* Bunge	4
*Polygala tenuifolia* Willd.	4
*Bupleurum falcatum* L.	4
*Gardenia jasminoides* Ellis	4
*Aucklandia lappa* Decne	2
*Glycyrrhiza uralensis* Fisch.	1.2
*Zingiber officinale* Roscoe	3
*Ziziphus jujuba* Mill.	3
Total amount	49.2 g

## Data Availability

The data presented in this study are not publicly available due to institutional data management policies but are available from the corresponding author upon reasonable request.
